# Is Thymic Involution Truly a Deterioration or an Adaptation?

**DOI:** 10.1007/s11538-025-01569-0

**Published:** 2026-01-17

**Authors:** Yoh Iwasa, Rena Hayashi, Akane Hara, Kosei Matsuo

**Affiliations:** 1 Department of Biology, Faculty of Science, Kyushu University, 744 Motooka, Nishi-Ku, Fukuoka, 819-0395 Japan; 2 Graduate School of Medicine and Pharmaceutical Sciences, University of Toyama, 2630, Sugitani, Toyama, 930-0194 Japan

**Keywords:** Thymic involution, Optimal rate of naïve T cell production, Peripheral pool of naïve T cells, Pontryagin’s maximum principle

## Abstract

In mammals, the immune system recognizes and combats pathogens while retaining a memory of prior encounters. In the thymus, naïve T cells capable of recognizing specific antigens are generated through random gene rearrangement, ensuring a diverse immune repertoire. However, the production rate of naïve T cells declines with age, typically following an exponential or power-law function—a phenomenon known as thymic involution, which is often regarded as a deterioration of biological function (immunosenescence). In this paper, we propose a novel theory suggesting that thymic involution may represent an adaptive strategy. As individuals age, repeated exposure to diverse pathogens leads to the accumulation of memory T cells, thereby reducing the need for newly generated naïve T cells to combat infections. Moreover, naïve T cells can persist in the periphery and retain the capacity to initiate immune responses against novel antigens. Using Pontryagin’s Maximum Principle, we calculate the optimal schedule of naïve T cell production. The results show that the production rate peaks during a brief period shortly after birth, followed by an exponential decline throughout life, eventually reaching a phase in which naïve T cell production ceases. If peripheral naïve T cells decay very slowly, the optimal strategy may consist of producing all cohorts at birth, with no subsequent production.

## Introduction

**Fig. 1:**
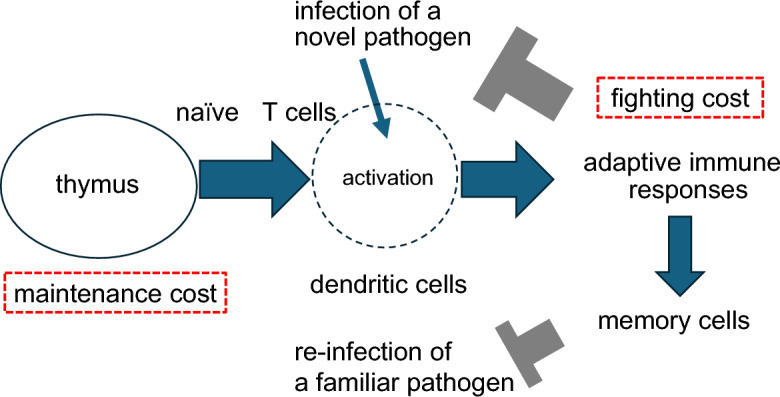
Scheme of the model. The model describes a tradeoff between fighting and maintenance costs, highlighted in the red boxes. As the host encounters infections by pathogens, memory cells accumulate, reducing the need to develop immunity to novel pathogens. Consequently, the production of naïve T cells in the thymus declines with age

Acquired immunity recognizes components of invading pathogens as foreign and triggers immune responses to defend against them (Parham 2021). In the thymus, T cells capable of recognizing diverse antigens are generated through proliferation and random gene rearrangement. Those that react to the body’s own components are eliminated through a process called negative selection. Through multiple rounds of selection and differentiation, a highly diverse population of naïve T cells is produced, each capable of recognizing a specific antigen. These naïve T cells migrate from the thymus to peripheral tissues, where they become activated upon encountering foreign antigens (Pennock et al. 2013; Minato et al. 2020). Once activated, they stimulate B cells, cytotoxic T cells, neutrophils, and other immune cells to eliminate pathogenic substances. Following the successful suppression of pathogens, some of these activated T cells differentiate into memory cells, which persist in the body and enable a rapid immune response upon future exposure to the same antigens (Fig. 1).

The supply rate of naïve T cells from the thymus declines with age, following an exponential decay over age (Steinmann et al. 1985; Douek et al. 1998; Lewkiewicz et al. 2019; de Boer et al. 2023). Between infancy and around 18 years of age, this rate drops to about one-tenth of its initial level (Steinmann et al. 1985; Kulesh et al. 2024). The thymic epithelial tissue responsible for T cell production begins to deteriorate as early as one year of age, even though the thymus continues to grow throughout childhood and reaches its maximum size at puberty. This decline is accompanied by a reduction in T-cell receptor excision circles (TRECs)—circular DNA fragments generated during TCR gene rearrangement that serve as markers of recent thymic emigrants (Lynch et al. 2009; de Barber et al. 2012). As a result, the diversity of peripheral naïve T cells diminishes with age, leading to slower immune responses against novel pathogens. This process, known as thymic involution, is considered a hallmark of immune aging, or immunosenescence (Sidler et al. 2013; Palmer et al. 2018), and results in age-dependent reductions in TCR repertoire diversity (Goronzy et al. 2015a; Hu et al. 2024). Kotsubo et al. (2025) analyzed age-dependent susceptibility to food allergies by modeling the decline in thymic naïve T cell output in conjunction with the Th2 and iTreg cell dynamics previously developed for pollen allergy by Hara and Iwasa (2017). Thymic involution has also been reported in nonhuman animals, such as rats and dogs, although the decline in thymic function occurs faster than in humans (Fujiwara et al. 2012; Appay and Sauce 2014).

In evolutionary biology, aging is understood as the outcome of the accumulation of deleterious mutations that exert harmful effects later in life. Mutations that negatively impact individuals during youth—especially before reproductive age—are efficiently eliminated by strong natural selection. In contrast, deleterious mutations that reduce survival after reproductive age are subject to much weaker selective pressure. Consequently, under mutation–selection balance, populations accumulate numerous mutations that manifest their detrimental effects in old age (Hamilton 1966; Charlesworth 2000; Shanley et al. 2009).

In this paper, we propose a novel theoretical framework suggesting that the age-related decline in naïve T cell production by the thymus represents an adaptive strategy. In natural environments, hosts are exposed to a wide variety of pathogens and accumulate memory T cells to defend against them as they age. Consequently, the necessity to generate new naïve T cells to combat unfamiliar pathogens diminishes over age. If thymic activity in producing naïve T cells is governed by a balance between the need for immunity against previously unencountered pathogens and the cost of maintaining thymic function, then a reduced need for novel immunity would render the decline in thymic activity adaptive for the host. After being released from the thymus, naïve T cells proliferate in the periphery, increasing their total number (de Boer et al. 2023). However, this increase in cell number does not enhance the diversity of naïve T cells, which is critical for both the speed of acquired immune responses against novel antigens and the suppression of pathogen proliferation. When the supply of naïve T cells from the thymus is low, the time required for specific naïve T cells to recognize a pathogen is prolonged, allowing the pathogen to proliferate and thereby increasing the cost of controlling the infection.

Naïve T cells may persist for extended periods in peripheral tissues such as lymph nodes, the spleen, and mucosa-associated lymphoid tissues (Tough and Sprent 1995; Ganusov and de Boer 2007; Baines et al. 2009; Rane et al. 2022). If naïve T cells have very long lifespans (i.e., slow turnover), the thymus could produce them all early in life, potentially rendering thymic function unnecessary in later years. Conversely, if naïve T cells in peripheral tissues have short lifespans (i.e., rapid turnover), continuous production by the thymus would be required.

Let $N\left( t \right)$ denote the number of naïve T cell clones in the peripheral tissues. We assume that $N\left( t \right)$, the number of naïve T cell clones in the periphery, determines the overall diversity of the naïve T cell population. $N\left( t \right)$ increases when naïve T cells are produced and released from the thymus. Let $h\left( t \right)$ represent the age-dependent production rate of new naïve T cells from the thymus; this rate is unaffected by the proliferation of naïve T cells in the periphery. Here, we track the number of clones of peripheral naïve T cells, rather than their total cell number. Let $u$ denote the exponential rate of decrease of the number of clones. Then, the dynamics of $N\left( t \right)$ are described by the differential equation: $\frac{dN}{{dt}} = h\left( t \right) - uN$.

For mathematical simplicity, we first consider the case in which the turnover of naïve T cell clones in the periphery is rapid. In this case, the dynamics quickly reach a quasi-stationary state, where $N\left( t \right) \approx \frac{1}{u}h\left( t \right)$ holds. Thus, the diversity of peripheral naïve T cell clones is primarily determined by the thymic production rate, $h\left( t \right)$. Our analysis shows that the optimal rate of novel naïve T cell production from the thymus should decline with age, following either an exponential or a power-law function, depending on the variability in encounter rates with different pathogen strains.

Later, we consider the dynamics of naïve T cell clones in the periphery, described by the differential equation of $N\left( t \right)$. The host aims to determine the optimal production rate of naïve T cells as a function of age, $t$. A useful mathematical tool for analyzing such control problems is Pontryagin’s Maximum Principle (PMP) (Pontryagin et al. 1962; Intriligator 1971). The key concept is to consider the ‘marginal value’ of each state variable, defined as the fitness effect of a unit increment in the state variable $N\left( t \right)$ at age $t$. Models analyzed using PMP include the scheduling of the switch from growth to reproduction (Léon 1976; Vincent and Pulliam 1980), the trade-off between annuals and perennials (Iwasa and Cohen 1989), lymphocyte populations in immunology (Perelson et al. 1976, 1978), and investment in defense chemicals such as alkaloids and terpenes (Iwasa et al. 1996, 2024, 2025). According to our analysis, the optimal rate of naïve T cell production, $h\left( t \right)$, exhibits the following age dependence: At birth, $h\left( t \right)$ reaches a peak production rate over a short initial interval (initial phase). Subsequently, $h\left( t \right)$ decreases exponentially with age (middle phase). At a certain age, $h\left( t \right)$ drops to zero, and no further naïve T cells are produced beyond this point (final phase). If the decay rate of naïve T cells in the periphery is very low, all naïve T cells are produced in a short period after birth, with no additional production thereafter.

## Model

Suppose there are many different antigens that are potentially pathogenic, which we denote as $i = 1,2,3,....,n$, where $n$ is the total number of antigens that a host encounters over its lifetime. The host encounters these antigens at random. Some are pathogenic and can invade the body and proliferate. However, others are not pathogenic, either because they cannot proliferate within the host body or because they are obstructed by the barrier functions of the skin and other epithelial tissues, or by the mechanisms of innate immunity. When the host encounters a pathogen that could not be suppressed by the innate immunity, it requires the actions of adaptive immunity to prevent harm. Let $f_{i}$ denote the rate of encounter with antigens of type $i$ that requires the action of adaptive immunity $\left( {i = 1,2,...,n} \right)$.

Acquired (adaptive) immunity is a mechanism through which immune cells specific to a particular antigen proliferate and mount a targeted response to fight the disease. After the infection is resolved, the host retains memory cells that can be activated if the same pathogen reinfects the host in the future. When a host encounters a pathogen and retains memory cells reactive to it, the host can respond to reinfection relatively quickly. However, if the host is infected by newly invaded pathogens, adaptive immunity must be activated. The probability that the antigen has not been encountered before is $exp\left[ { - \int\limits_{0}^{t} {f_{i} dt} } \right] = e^{{ - f_{i} t}}$, where $t$ is the age of the host.

The process of combating a novel pathogen begins with the activation of specific naïve T cells by dendritic cells in peripheral tissues. This is followed by the activation of various immune cells, including B cells and cytotoxic T cells, which collectively coordinate the immune response. We assume that the novel pathogen will eventually be suppressed, resulting in the formation of memory cells that can rapidly respond to future reinfections.

As explained in an earlier section, $N\left( t \right)$ denotes the number of naïve T cell clones in the periphery. This number increases when naïve T cells are produced and released from the thymus. We assume that $N\left( t \right)$, the number of naïve T cell clones in the periphery, determines the overall diversity of the naïve T cell population. Specifically, we assume that the cost for fighting a novel pathogen is inversely proportional to the number of peripheral naïve T cell clones, i.e., $K\frac{1}{N\left( t \right)}$, where the proportionality coefficient $K$ represents the magnitude of harm caused by infection of a novel pathogen. This particular function was chosen as the simplest representation of the underlying idea.

The amount of cost the host must pay in suppressing the pathogens that encounter within a short time interval of length ${\Delta }t$ is the cost of fighting a single novel pathogen multiplied by the probability of encountering a novel pathogen that requires the activation of adaptive immunity: $\sum\limits_{i = 1}^{n} {f_{i} {\Delta }t \cdot e^{{ - f_{i} t}} \cdot K\frac{1}{N\left( t \right)}}$, which indicates "fighting cost." This decreases with the number of clones of peripheral naïve T cells $N\left( t \right)$.

We also consider the cost for maintaining thymic activity. In the thymus, the training of immune cells takes place. To generate a large diversity of antigen-recognizing sites, developing T cells undergo gene rearrangement (i.e., V(D)J recombination), resulting in a diverse repertoire of T-cell receptors (TCRs). Following this, a "negative selection" procedure occurs, during which cells that recognize the body’s own proteins and other materials are eliminated. Developing T cells undergo multiple cycles of proliferation and gene rearrangement, interspersed with negative selection, ultimately giving rise to a population of naïve T cells with a highly diverse range of antigen specificities. Hence, maintaining the thymus, which continues to produce naïve T cells, involves a significant biological cost. For simplicity, we assume that it is directly proportional to $h\left( t \right)$ with a proportionality constant of $m$. Therefore, the cost of maintaining the rate of naïve T cell supply is $mh\left( t \right) \cdot {\Delta }t$.

Combining the costs of engaging in defense and maintaining readiness, the total cost associated with the defensive action per unit time is given by:

1 $${\Psi }\left( t \right) = \sum\limits_{i = 1}^{n} {f_{i} \cdot e^{{ - f_{i} t}} \cdot K\frac{1}{N\left( t \right)} + mh\left( t \right) \to {\mathrm{minimum}}}$$

The first and second terms on the right-hand side represent the fighting cost and maintenance cost per unit time, respectively.

We assume the following differential equation for $N\left( t \right)$:

2 $$\frac{dN}{{dt}} = h\left( t \right) - uN\;for\;0 < t < T$$

for 0 < *t* < *T*. The initial condition is $N\left( 0 \right) = 0$. If decay rate $u$ is large, maintaining a certain level of naïve T cell clones requires continuous production by the thymus. Conversely, if $u$ is small, the thymus can produce most of the naïve T cells at birth and cease production thereafter. Therefore, the temporal pattern of naïve T cell production from the thymus should critically depend on the magnitude of the decay rate (Table 1).

**Table 1:** Symbols used in the models of this study

$h\left( t \right)$	production rate of naïveT cells
$N\left( t \right)$	number of naïve T cell clones in the periphery
$t$	age
$T$	maximum age
$f_{i}$	encounter rate with pathogen strain $i$
$n$	number of pathogen strains a single host encounters over its lifetime
$\phi$	the fitness, the quantity to maximize
$\overline{f}$	mean value of encounter rate $f_{i}$
$a$ and $b$	two parameters characterizing the Gamma distribution of $f$
$K$	magnitude of fighting cost
$m$	production cost of novel naïve T cells
$u$	decay rate of naïve T cell clones in the periphery
$h_{max}$	maximum rate of naïve T cell production
$\lambda \left( t \right)$	costate variable, indicating the increase of $\phi$ caused by unit increase in $N\left( t \right)$

## Optimal Production Rate of Naïve T cells Under Rapid Turnover

For mathematical simplicity, we first examine the case in which the turnover of naïve T cells in the periphery is rapid. In this case, the state variable $N\left( t \right)$ quickly reaches a quasi-stationary state, where the approximate relationship, $N\left( t \right) \approx \frac{1}{u}h\left( t \right)$ holds. This implies that the peripheral diversity of naïve T cells (i.e., the number of clones) is primarily determined by the thymic production rate. Under this assumption, the quantity ${\Psi }\left( t \right)$ defined by Eq. (1) becomes

3 $${\Psi }\left( t \right) = \sum\limits_{i = 1}^{n} {f_{i} \cdot e^{{ - f_{i} t}} \cdot K\frac{u}{h\left( t \right)} + mh\left( t \right) \to {\mathrm{minimum}}}$$

which is to be minimized at each age $t$ (for $0 < t < T$). The optimal value of $h\left( t \right)$ is the one that minimizes ${\Psi }\left( t \right)$, at each $t$. This can be calculated from setting the derivative equal to zero:

$$0 = \partial {\Psi }/\partial h = \sum\limits_{i = 1}^{n} {f_{i} e^{{ - f_{i} t}} Ku\frac{ - 1}{{h\left( t \right)^{2} }} + m}$$

which leads to

4 $$h\left( t \right) = \sqrt {\frac{Kun}{m} \cdot \frac{1}{n}\sum\limits_{i = 1}^{n} {f_{i} e^{{ - f_{i} t}} } }$$

We can interpret $K$ and $m$ as the costs of fighting and maintenance, respectively. $n$ is the expected number of antigens that are potentially pathogenic. The term $\frac{1}{n}\sum\limits_{i = 1}^{n} {f_{i} e^{{ - f_{i} t}} }$ is the quantity $f_{i} e^{{ - f_{i} t}}$ averaged over different antigens. The optimal rate of naïve T cell supply from the thymus $h\left( t \right)$ given by Eq. (4) is a decreasing function of age $t$. This may provide an explanation for thymic involution. In the following two sections, we examine the decrease of $h\left( t \right)$ with age, and how the pattern of decrease may vary with $t$ depending on parameters.

We start to consider the case in which different antigens have the same $f_{i}$, the rate of encounter with pathogens in the environment that require action of adaptive immunity. We denote $f_{i} = \overline{f}$, for $i = 1,2,...,n$. The formula for the optimal $h\left( t \right)$ given by Eq. (4) becomes as follows:

5a $$h\left( t \right) = \sqrt{\frac{Kun}{m}} \cdot \left( {\overline{f}e^{{ - \overline{f}t}} } \right)^{\frac{1}{2}} = \sqrt {\frac{{Kun\overline{f}}}{m}} \cdot e^{{ - \frac{{\overline{f}}}{2}t}}$$

This implies that the optimal rate of naïve T cell supply decreases with age $t$ following an exponential function of age $t$. The number of clones of peripheral naïve T cells is given as follows:

5b $$N\left( t \right) = \frac{1}{u}h\left( t \right) = \sqrt {\frac{{Kn\overline{f}}}{mu}} \cdot e^{{ - \frac{{\overline{f}}}{2}t}}$$

which decreases with age exponentially.

### Parameter Dependence

We here discuss the parameter dependence of the optimal rate of naïve T cell production in the thymus. Even if their exact values are currently not available, estimating them will help assess the applicability of the model.

The initial rate of naïve T cells is $\sqrt {\frac{{Kun\overline{f}}}{m}}$, which is high when encountering a new pathogen is risky (large $K$), cost of maintaining the thymus is small (small $m$), and the total rate of encounter with pathogen strains in the environment is high (large $n\overline{f}$).

The rate of decrease in $h\left( t \right)$ with age is fast if the rate of encountering with pathogens is high (large $\overline{f}$). This is because the host acquires immune memory quickly in the environment where each pathogen is abundant. The formula Eq. (5a) predicts that thymic involution occurs rapidly in unsanitary environments and more slowly in hygienic environments.

The total amount of naïve T cell production is expressed as follows:

6 $$\mathop \smallint \limits_{0}^{T} h\left( t \right)dt \approx \sqrt {\frac{{Kun\overline{f}}}{m}} \cdot \mathop \smallint \limits_{0}^{\infty } e^{{ - \frac{{\overline{f}}}{2}t}} dt = 2\sqrt {\frac{Kun}{{m\overline{f}}}}$$

where we approximate the maximum age by infinity, which is an acceptable approximation because the rate of supply in naïve T cells decline quickly with age. Equation (6) indicates that the total lifetime number of naïve T cell clones formed increases with the cost of fighting a novel pathogen infection and the number of pathogens, while decreasing with the cost of maintaining thymic activity and the rate of encountering each strain of risky pathogens.

**Fig. 2:**
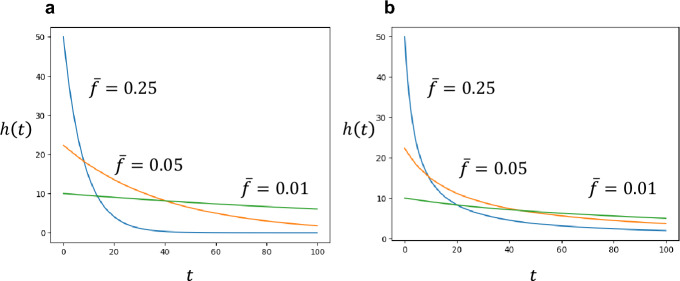
Optimal schedule of naïve T cell production $h\left( t \right)$. The horizontal axis indicates age $t$. **a** When all strains have the same encounter rate $\overline{f}$. Different curves correspond to the cases with different $\overline{f}$, given by Eq. (8a) in the text. A larger $\overline{f}$ makes the initial level $h\left( 0 \right)$ larger, decreases more rapidly, and the total production of naïve T cells (area under the curve) larger. **b** When the encounter rate $f$ has a large variance between strains, given by an exponential distribution (Gamma-distribution with $a = 1$). $h\left( t \right)$ decreases with age $t$, following Eq. (8b), which is a power function. The other parameter is: $Kun/m = 10^{4}$

(i) Rate of encountering pathogens in the environment: Fig. 2a illustrates how the optimal rate of naïve T cells declines with the age for several different values of encounter rate $\overline{f}$. If we compare areas with the same number of strains ($n$ is the same), as the rate of encountering with each strain $\overline{f}$ is larger, the optimal rate of naïve T cell production $h\left( t \right)$ starts higher, but it decreases more quickly with age; and the total number of naïve T cell clones to produce is smaller.

(ii) Ratio of fighting cost to maintenance cost: We note that the optimal rate of naïve T cell production is enhanced when the risk of the fighting $K$ is high and the cost of maintaining the ability to produce naïve T cells $m$ is small. When their ratio $\frac{K}{m}$ increases, the rate of naïve T cell production increases for all ages. Hence, the total number of naïve T cell clones to produce in lifetime also increases in proportion to the square-root of the ratio $\frac{K}{m}$.

(iii) Number of pathogen strains: The dependence of $h\left( t \right)$ on $n$, the number of pathogen strains, is the same as its dependence on $\frac{K}{m}$. Hence, we conclude that the optimal rate of naïve T cell production is higher for all ages when there are more pathogen strains.

## When Antigens Differ in Encounter Frequency

Quantity $f_{i}$ indicates the rate of encountering with the antigen that requires host’s adaptive immunity operation. This may differ between antigens. The sum given by Eq. (4) is a mixture of exponential functions with different rates of decrease. In this section, we consider the effect of differences in $f_{i}$ among pathogen strains.

If we plot $logh\left( t \right)$ over age $t$, it is represented by a curve with a negative slope and the rate of decrease is faster for young ages and becomes slower for older ages. This is proved mathematically because $\frac{d}{dt}logh\left( t \right) < 0$ and $\frac{{d^{2} }}{{dt^{2} }}logh\left( t \right) \ge 0$ hold, with equality only when all $f_{i}$ are exactly the same. Refer to Appendix A for the derivation.

### Gamma Distribution

We consider the case that the distribution of $f_{i}$ can be approximately by a continuous probability distribution of $f$, denoted by $\psi \left( f \right)$. Suppose that $\psi \left( f \right)$ is close to a gamma distribution $\psi \left( f \right) = f^{a - 1} e^{ - bf} /\left( {{\Gamma }\left( a \right)\frac{1}{{b^{a} }}} \right)$, which is a probability distribution over positive value ($f > 0$) with a single peak (if $a > 1$) or the one with a decreasing probability density (if $0 < a \le 1$). It has mean $\overline{f} = \frac{a}{b}$ and variance $\frac{a}{{b^{2} }}$. Then, we have the following approximation:

$$\begin{gathered} \frac{1}{n}\mathop \sum \limits_{i = 1}^{n} f_{i} e^{{ - f_{i} t}} \approx \mathop \smallint \limits_{0}^{\infty } fe^{ - ft} \cdot \psi \left( f \right)df = \mathop \smallint \limits_{0}^{\infty } fe^{ - ft} \cdot \frac{{f^{a - 1} e^{ - bf} }}{{{\Gamma }\left( a \right)\frac{1}{{b^{a} }}}}df \hfill \\ = \frac{a}{b}\frac{1}{{\left( {1 + \frac{t}{b}} \right)^{a + 1} }} = { }\overline{f}\frac{1}{{\left( {1 + \frac{{\overline{f}t}}{a}} \right)^{a + 1} }} \hfill \\ \end{gathered}$$

In the last step, we used that the mean value of $f$ is $\overline{f} = \frac{a}{b}$. Hence, the optimal rate of naïve T cell production, given by Eq. (4), becomes as follows:

7 $$h\left( t \right) = \sqrt {\frac{{Kun\overline{f}}}{m}} \cdot \frac{1}{{\left( {1 + \frac{{\overline{f}t}}{a}} \right)^{{\frac{a + 1}{2}}} }}$$

**Fig. 3:**
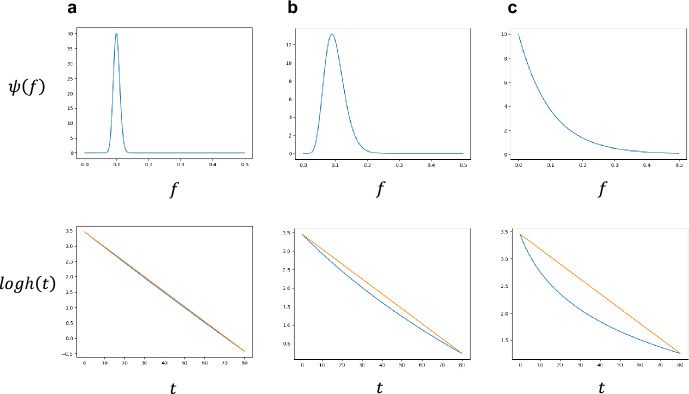
Optimal schedule of naïve T cell production $h\left( t \right)$ for different probability distribution of $f$. The encounter rate $f$ of different strains follows a Gamma-distributions with probability density $\psi \left( f \right)$. Three cases, (a), (b), and (c), have the same mean encounter rate $\overline{f} = b/a = 0.1$. They however differ in variance of $f$. Ratio, $variance/mean^{2} = 1/a$, is as follows: **a** $0.01$; **b** $0.1$; **c** $1$. The upper panels show the probability density $\psi \left( f \right)$, while the lower panels depict the logarithm of the optimal production rate, $logh\left( t \right)$. The horizontal axis represents age. Parameters are: $Kun/m = 10^{4}$, and **a** $a = 100, b = 1000$. **b** $a = 10, b = 100$. **c** $a = 1, b = 10$

The ratio of variance to the squared mean, given by $variance/mean^{2} = 1/a$ provides a good measure for the relative magnitude of variation in $f$. The optimal rate of naïve T cell supply $h\left( t \right)$ becomes simplified forms in two different choices of $a$, as shown below:

(i) When $a$ is very large: In the limit of $a \to \infty$, we have the following result:

8a $$h\left( t \right) = \sqrt {\frac{{Kun\overline{f}}}{m}} \cdot e^{{ - \frac{{\overline{f}}}{2}t}}$$

because $lim_{n \to \infty } \left( {1 + \frac{1}{n}} \right)^{n} = e$. Equation (8a) reduces to Eq. (5a), which corresponds to the case where different antigens have identical values of $f_{i}$.

(ii) When $a$ is equal to unity: By setting $a = 1$, the gamma distribution becomes an exponential distribution with mean $\overline{f}$ (i.e. $\psi \left( t \right) = e^{{ - f/\overline{f}}} \left( {1/\overline{f}} \right)$). Then we have the following simple formula:

8b $$h\left( t \right) = \sqrt {\frac{{Kun\overline{f}}}{m}} \cdot \frac{1}{{1 + \overline{f}t}}$$

which decreases as a power function (or a hyperbolic function) of age $t$. This corresponds to the case where the values of $f_{i}$ of different antigens approximately follow an exponential distribution with mean $\overline{f}$ and large variance. An example is illustrated in Fig. 2b.

(iii) When $1 < a < \infty$: We have an intermediate situation between exponential and power functions. In Fig. 3, three columns, (a), (b), and (c), illustrate the cases with $a = 100$, $a = 10$, and $a = 1$, respectively. Top parts indicate distribution of $f$. Figure 3a, b, and c have the same mean $\overline{f} = \frac{a}{b}$ but different variance $\frac{a}{{b^{2} }} = \overline{f}^{2} \frac{1}{a}$. The variance is small for Fig. 3a, intermediate for Fig. 3b, and large for Fig. 3c. Note that in Fig. 3c the probability distribution of $f$ is an exponential distribution. Bottom parts indicate $logh\left( t \right)$, the optimal supply rates in logarithmic scale. We can see that the slope of $logh\left( t \right)$ is negative for all ages, implying that $h\left( t \right)$ decreases with age. The rate of decrease in $logh\left( t \right)$ is faster for young ages and becomes slower for older ages. Hence the curve of $logh\left( t \right)$ has a positive second derivative as a function of $t$, which can be shown mathematically (see Appendix A).

In ecology, a "community" refers to an assembly of different species that inhabit a specific area and coexist while interacting with one another. A commonly observed species-abundance relation in a well-studied community is a lognormal distribution (Preston 1948; Pielou 1966; Whittaker 1975). It states that the fraction of species with the logarithmic abundance $z = logf$ has a normal distribution with mean $\mu$ and variance $\sigma^{2}$.

In Appendix A, we examine the optimal rate of naïve T cell supply from the thymus. When comparing distributions with the same mean value $\overline{f} = exp\left[ {\mu + \sigma^{2} /2} \right]$, but different variances, the naïve T cell supply $h\left( t \right)$ decreases with age $t$. When $\sigma^{2}$ is small, the distribution of $f$ is sharpy centered around $\overline{f}$, and the optimal $h\left( t \right)$ is close to an exponential function, indicated by a straight line. As $\sigma^{2}$ becomes larger, the distribution of $f$ has a larger variance, and $h\left( t \right)$ becomes more like a power function of age. The curve of $logh\left( t \right)$ is deviated from a straight line more strongly as variance $\sigma^{2}$ increases.

## Dynamic Optimization Incorporating Peripheral Naïve T Cell Clone Behavior

Naïve T cells released from the thymus may proliferate, without changing the number of clones in the periphery. Concerning the time change of the number of clones of peripheral naïve T cells, which affects the efficiency of coping with novel pathogen strains, we consider the differential equation of $N\left( t \right)$, the number of naïve T cell clones in the periphery. $h\left( t \right)$ denotes the rate of naïve T cell production by the thymus, representing the rate of increase in the number of peripheral clones. $u$ represents the exponential decay rate of naïve T cell clones in the periphery, and $t$ denotes the age. We assume the differential equation for $N\left( t \right)$ given as Eq. (2) with the initial condition $N\left( 0 \right) = 0$.

The optimal production rate of naïve T cell from the thymus reflects a trade-off between fighting and maintenance costs, which are represented by the first and second terms of Eq. (1), respectively. If the turnover of naïve T cells is not very rapid, we must compare the costs incurred at different times, because rapid production at age $t$ increases the number of peripheral naïve T cell clones after age $t$. To combine the effects at different ages $t$, we use a time integral. Instead of focusing on cost minimization, we consider fitness optimization, as is common in most theoretical studies of life-history strategies. We consider the following quantity: $\phi = C - \int\limits_{0}^{T} {{\Psi }\left( t \right)dt}$, as a measure of advantage, where $C$ is the fitness level in the absence of costs associated with naïve T cell production in the thymus and the cost arising from the delay in developing acquired immunity due to the scarcity of naïve T cells. From Eq. (1), we have the following:

9 $$\phi = C - \mathop \smallint \limits_{0}^{T} \left( {\frac{K}{{N\left( {t^{\prime } } \right)}}\mathop \sum \limits_{i = 1}^{n} f_{i} e^{{ - f_{i} t^{\prime } }} + mh\left( {t^{\prime } } \right)} \right)dt^{\prime } \to {\mathrm{maximum}}$$

We seek the optimal rate of naïve T cell production from the thymus, $h\left( t \right)$, that maximizes $\phi$ under the constraint of the differential Eq. (2) and $0 \le h\left( t \right) \le h_{max}$, where $h_{max}$ is the maximum production rate. This scenario represents a typical control problem.

The control problem is analyzed using Pontryagin’s maximum principle (Pontryagin et al. 1962; Intriligator 1971). We define the Hamiltonian as follows:

10 $$H = - \left( {\frac{K}{N\left( t \right)}\mathop \sum \limits_{i = 1}^{n} f_{i} e^{{ - f_{i} t}} + mh\left( t \right)} \right) + \lambda \left( t \right)\left( {h\left( t \right) - uN} \right)$$

where $\lambda \left( t \right)$ is a costate variable indicating the marginal value of the naïve T cell abundance in the periphery $N\left( t \right)$. The time change of the costate variable satisfies the following differential equation:

11 $$\frac{d\lambda }{{dt}} = - \frac{\partial H}{{\partial N}} = - \frac{K}{{N\left( t \right)^{2} }}\sum\limits_{i = 1}^{n} {f_{i} e^{{ - f_{i} t}} + u\lambda \left( t \right)}$$

with the terminal condition $\lambda \left( T \right) = 0$.

Given that an optimal control and corresponding state solution exists, Pontryagin’s maximum principle states that the control variable $h\left( t \right)$ must be chosen to maximize the Hamiltonian at each age $t$. Since the Hamiltonian depends on the control variable $h\left( t \right)$ as follows:

$$H = \left( { - m + \lambda \left( t \right)} \right)h\left( t \right) + \left[ {{\text{terms independent of}}\, h} \right],$$

the optimal control satisfies the following relationship:

12a $${\mathrm{If}}\;\lambda \left( t \right) > m,\;h\left( t \right) = h_{max}.$$

12b $${\mathrm{Only}}\;{\mathrm{if}}\;\lambda \left( t \right) = m,\;0 \le h\left( t \right) \le h_{max},$$

12c $${\mathrm{If}}\;\lambda \left( t \right) < m,\;h\left( t \right) = 0.$$

We aim to construct a candidate solution that satisfies all the conditions specified by Pontryagin’s maximum principle. Mathematically, this principle provides a set of necessary conditions for optimal control. However, based on our experiences applying this method to various biological problems, a candidate solution that aligns with biological reasoning is typically the optimal control solution and often yields novel insights.

### When Strains Have the Same Encounter Rate

**Fig. 4:**
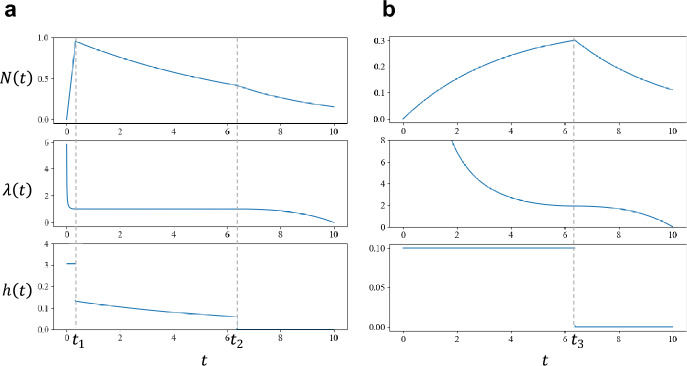
Optimal schedule of naïve T cell production. **a** Three-phase solution. Top, middle, and bottom panels indicate $N\left( t \right)$, $h\left( t \right)$, and $\lambda \left( t \right)$, respectively. The horizontal axis indicates age $t$. The production rate is the maximum in the initial phase ($0 < t < t_{1}$); an intermediate value decreasing with age exponentially in the middle phase ($t_{1} < t < t_{2}$), which is a singular control; zero in the final phase ($t_{2} < t < T$). **b** Two-phase solution. Middle phase with singular control does not exist. The switching from the initial phase (maximum production rate) to the final phase (no production) occurs at $t_{3}$. Parameters are: **a** $n = 1000$, $\overline{f} = 0.275$, $m = 1$, $K = 0.001$, $h_{max} = 3$, $T = 10$; **b** $n = 1000,$ $\overline{f} = 0.2751$, $m = 1$, $K = 0.001$,$h_{max} = 0.1$, $T = 10$

We first focus on the situation where different strains have an equal encounter rate: $f_{i} \approx \overline{f}$ ($i = 1,2,3,..,n$). Then, $\sum\limits_{i = 1}^{n} {f_{i} e^{{ - f_{i} t}} \approx n\overline{f}e^{{ - \overline{f}t}} }$ holds, where $n$ is the number of pathogen strains and $\overline{f}$ is their common rate of encounter in the environment. Figure 4a illustrates a typical solution of the control problem. The top, middle, and bottom panels indicate the optimal schedule of naïve T cell production $h\left( t \right)$, the number of naïve T cell clones in the periphery $N\left( t \right)$, and the costate variable $\lambda \left( t \right)$, respectively. The horizontal axis represents age $t\left( {0 < t < T} \right)$. There are three qualitatively different phases. In contrast, Fig. 4b illustrates the solution with two different phases.

(i) Three-phase solution: If Eq. (12b) holds in an interval of a positive length, control variable $h\left( t \right)$ is an intermediate value and $\lambda \left( t \right) = m$ holds identically. This is an example of singular control (Kelley et al. 1967; Robbins 1967). Singular control arises in many dynamic optimization problems when the Hamiltonian is a linear function of the control variables, and it constitutes an important aspect of life history theory (King and Roughgarden 1982; Iwasa and Roughgarden 1984; Irie and Iwasa 2005; Ioslovich and Gutman 2005; Iwasa et al. 2025).

As a candidate for the optimal control schedule, we consider the following three-phase solution (Fig. 4a):

Initial phase $\left( {0 < t < t_{1} } \right)$: Naïve T cells are produced at the maximum rate.

Middle phase $\left( {t_{1} < t < t_{2} } \right)$: Naïve T cells are produced at an intermediate rate, and singular control occurs.

Final phase $\left( {t_{1} < t < t_{2} } \right)$: No naïve T cells is produced.

The detailed procedure for constructing the candidate solution is explained in Appendix B. The solution of this type can be constructed by first noting the singular control in the middle phase $\left( {t_{1} < t < t_{2} } \right)$. From $\lambda \left( t \right) = m$ and the simplifying assumption: $f_{i} \cong \overline{f}$, Eq. (11) becomes $0 = - \frac{K}{{N\left( t \right)^{2} }}n\overline{f}e^{{ - \overline{f}t}} + um$, which is rewritten as follows:

13a $$N\left( t \right) = \sqrt {\frac{{Kn\overline{f}}}{mu}} e^{{ - \frac{{\overline{f}}}{2}t}}$$

From Eq. (2), we have the rate of naïve T cell production from the thymus as follows:

13b $$h\left( t \right) = \frac{dN}{{dt}} + uN = \left( {u - \frac{{\overline{f}}}{2}} \right)\sqrt {\frac{{Kn\overline{f}}}{mu}} e^{{ - \frac{{\overline{f}}}{2}t}}$$

It is very interesting to compare these values along the singular control with the corresponding values obtained from the simple non-dynamic optimization model studied in the previous sections. The number of clones of peripheral naïve T cells $N\left( t \right)$ along the singular control given by Eq. (13a) is the same as that given by Eq. (5b), which was obtained from the simple non-dynamic optimization model. In contrast, the production rate from the thymus $h\left( t \right)$ given by Eq. (13b) is smaller than Eq. (5a).

In the initial phase, naïve T cells are produced at the fastest rate: $h\left( t \right) = h_{max}$. The naïve T cell population starts from the initial condition $N\left( 0 \right) = 0$, increases rapidly, and reaches the level specified by the singular control, given by Eq. (13a) at age $t_{1}$. According to the calculation in Appendix B, we have the following equation for $t_{1}$:

14 $$\frac{{h_{max} }}{u}\left( {1 - e^{{ - ut_{1} }} } \right) = \sqrt {\frac{{Kn\overline{f}}}{mu}} e^{{ - \frac{{\overline{f}}}{2}t_{1} }}$$

In the final phase, no naïve cells are produced from the thymus. Near the final time, the marginal value $\lambda \left( t \right)$ is smaller than $m$ because the terminal condition gives $\lambda \left( T \right) = 0$. By integrating differential equation for $\lambda \left( t \right)$ backward from $t = T$, we can obtain the time at which $\lambda \left( t \right)$ reaches $m$, which determine $t_{2}$, time for the onset of the final phase, as follows:

15 $$T - t_{2} = \frac{1}{{u - \overline{f}}}log\left( {2 - \frac{{\overline{f}}}{u}} \right)$$

The derivation is explained in Appendix B. Equation (15) indicates that the length of the final phase, becomes longer as $u$ decreases.

If the values of $t_{1}$ from Eq. (14) and $t_{2}$ from Eq. (15) satisfy $t_{1} < t_{2}$, we can construct the three-phase solution, as illustrated in Fig. 4a.

There exists a second-order condition that must be satisfied during the phase of singular control, known as the generalized Legendre–Clebsch condition (Kelley et al. 1967; Robbins 1967; Iwasa and Roughgarden 1984). For the model in this study, we confirm that this condition is satisfied, as explained in Appendix C.

(ii) Two-phase solution: If the initial and final phases are long, and $t_{1}$ and $t_{2}$, obtained from Eqs. (14) and (15), respectively, satisfy $t_{1} \ge t_{2}$. Then, the middle phase (singular control) disappears. We denote $t_{3}$ as the date for switching between initial and final phases. We obtain the two-phase solution, as illustrated in Fig. 4b is given as follows:

Initial phase ($0 < t < t_{3}$): The naïve T cells are produced at the fastest rate: $h\left( t \right) = h_{max}$.

Final phase ($t_{3} < t < T$): No new naïve T cell is produced: $h\left( t \right) = 0$.

### When Strains Differ in Encounter Rate

We have focused the analysis on cases where different pathogen strains have the same encounter rate. If antigen strains differ in their frequency of encounter in the environment, we need to consider the distribution of pathogens based on their encounter rates. Let us consider the case in which the total number of antigens that the host is expected to encounter in its lifetime, $n$, is large and the distribution of $f_{i}$ ($i = 1,2,...,n$) can be approximated as sampled from a Gamma distribution. with the probability density: $\psi \left( f \right) = f^{a - 1} e^{ - bf} /\left( {{\Gamma }\left( a \right)\frac{1}{{b^{a} }}} \right)$, where ${\Gamma }\left( a \right)$ is a gamma function. In addition, if $u$ is large compared with $\overline{f}$, the solution includes middle phase where the singular control holds. According to the analysis in Appendix D, the number of clones of naïve T cells in the periphery and the production rate from the thymus are given as follows:

16a $$N\left( t \right) = \sqrt {\frac{{Kn\overline{f}}}{mu}} \frac{1}{{\left( {1 + \frac{{\overline{f}{ }t}}{a}} \right)^{{\frac{a + 1}{2}}} }}$$

16b $$h\left( t \right) = \sqrt {\frac{{Kn\overline{f}}}{mu}} \left( {u - \frac{{\frac{a + 1}{2} \cdot \overline{\frac{f}{a}} }}{{1 + \frac{{\overline{f}{ }t}}{a}}}} \right)\frac{1}{{\left( {1 + \frac{{\overline{f}{ }t}}{a}} \right)^{{\frac{a + 1}{2}}} }}$$

for $t_{1} < t < t_{2}$. The peripheral population of naïve T cells decreases as a power function of age. Refer to Appendix D for details.

## Discussion

Most literature on thymic involution considers it an example of age-related deterioration of immune function, often referring to it as "immunosenescence" (Sidler et al. 2013; Palmer et al. 2018). The term *senescence* is appropriate for describing the decline in immune function that occurs after an individual reaches reproductive age, as aging is generally characterized by a progressive decline in organ or tissue function due to the accumulation of cellular damage (Sun et al. 2023). However, this term is not suitable for decreases in biological function that occur from infancy to adolescence, as these changes precede reproductive maturity.

In this study, we examine thymic involution—represented by the age-dependent decline in the supply rate of naïve T cells—as an adaptive life history strategy. The reduction in the ability to produce naïve T cells is viewed as an optimal response to the decreasing need to cope with novel antigens, a need that naturally diminishes as immune memory accumulates through repeated exposures to infectious agents. Consequently, the optimal level of the thymus’s ability to produce naïve T cells decreases with age, following either an exponential or power-law function, depending on the variability in encounter rates among strains.

Naïve T cells remain in peripheral tissues for a certain period and retain the ability to generate new adaptive immune responses upon encountering novel foreign antigens (Tough and Sprent 1995; Pennock et al. 2013; Minato et al. 2020). To examine how the decline in production rate depends on the decay rate of naïve T cell diversity in the peripheral tissues, we considered the number of naïve T cell clones, $N\left(t\right)$, which is described by the differential equation in Eq. (2).

### When the Turnover of Peripheral Naïve T cells is Rapid

If the decay rate of naïve T cells is rapid, $N\left(t\right)$ is controlled by the production rate from the thymus, and a simple model that optimizes Eq. (3) separately at each time point is appropriate for discussing the age-dependent changes in thymic activity. The results are as follows:

First, when pathogen strains are similar in encounter rate, the optimal production rate of naïve T cells should decline exponentially with age. Several studies support this prediction of exponential decline (Steinmann et al. 1985; Bar-Dayan et al. 1999; Kulesh et al. 2024). Although cell proliferation may increase the total number of naïve T cells in the periphery, it does not enhance their antigenic diversity. The ability to detect novel antigens (pathogens) depends on the diversity of naïve T cells, which is determined by the number of newly formed naïve T cells produced by the thymus. The exponential decline in naïve T cell output from the thymus begins early in life (Douek et al. 1998; Kulesh et al. 2024), with the rate of decline slowing down at later ages (Ritter and Palmer 1999; Steinmann 1986; Mitchell et al. 2010).

Second, as the rate of pathogen encounters in the environment increases, the optimal production rate of naïve T cells at birth becomes higher; however, this rate declines more rapidly with age. As a result, the total number of naïve T cell clones produced over a lifetime would be smaller. It would be worthwhile to search for empirical evidence supporting or refuting this prediction. Thymic function in producing naïve T cells continues even into middle and older ages (Bar-Dayan et al. 1999).

Third, if the cost of fighting a novel pathogen through adaptive immunity is high, and the cost of maintaining thymic function to produce naïve T cells is low, then the production rate of naïve T cells increases at all ages. The same prediction applies when the number of pathogen strains in the environment is higher.

Fourth, if pathogen strains differ markedly in their encounter rates with the host, the optimal rate of naïve T cell production decreases as a power function rather than an exponential function of age. This prediction is supported by studies showing that the exponential rate of decline in naïve T cell supply is faster during early life and slows with age (Ritter and Palmer 1999; Steinmann 1986; Mitchell et al. 2010), suggesting a power-law relationship with age.

### If Naïve T Cells Survive in the Periphery for an Extended Period

If the decay rate of the peripheral naïve T cell population is slow, a dynamic optimization model—formalized by Eqs. (2) and (9)—is required. This model can be analyzed using Pontryagin’s Maximum Principle.

If the decay rate is not very slow compared to the encounter rate with pathogens, the singular control solution dominates the middle phase of the optimal strategy. The optimal number of clones of naïve T cells during this singular-control phase, as it changes with age, matches the value predicted by a simpler model that does not account for the peripheral pool of naïve T cells. In the singular-control solution, naïve T cell production exhibits a sharp peak shortly after birth, followed by a phase of lower but sustained production, and finally ends with a phase of no naïve T cell production from the thymus. Active production of naïve T cells in the thymus can be detected through T cell receptor excision circles (TRECs), circular DNA fragments that indicate gene rearrangement has occurred (Lynch et al. 2009; den Braber et al. 2012).

If the decay rate $u$ is very small, all naïve T cells are produced at the beginning of life, after which naïve T cell production ceases. The singular control phase disappears (Please refer to Appendix B for more details).

However, in humans, evidence supports the former pattern, showing that thymic production of naïve T cells continues into middle and older ages (Bar-Dayan et al. 1999). Studies on thymic development in species such as zebrafish, medaka, and others (Bajoghli et al. 2019) have concluded that thymic involution occurs in these species as well as in commonly studied animals like humans, mice, and rats. Thymic involution occurs in both humans and mice, but its consequences differ. Mice, with their short lifespan, rely primarily on continuous thymic output to replenish naive T cells. In contrast, humans experience a rapid decline in thymic activity after puberty, but naive T cells are maintained for decades in the periphery through survival signals and homeostatic proliferation (den Braber et al. 2012; Goronzy et al. 2015b; Lynch et al. 2009; Palmer 2013; Thomas et al. 2020). A more careful examination is needed regarding the existence of the singular-control phase, which lies between the initial peak period of naïve T cell production just after birth and the final cessation phase.

### When Pathogen Strains Differ in the Encounter Rate in the Environment

Pathogen strains are likely to differ in their encounter rates in the environment. The optimal rate of naïve T cell production then becomes the sum of multiple exponential functions, each with a different rate of decline. By approximating the distribution of encounter rates $f$ among strains using a probability distribution with a smooth density—such as a Gamma distribution—we can show that the sum of many strains with diverse decline rates is approximately equivalent to a power function, as expressed in Eq. (7). We can distinguish an exponential function from a power function by plotting their logarithmic values against age $t$ on the horizontal axis. For an exponential function, the plot fits a straight line, whereas a power function—or a mixture of multiple exponential functions—appears as a curve with a negative slope and a concave shape (positive second derivative). This curve exhibits a rapid decrease at small $t$ and a slower decrease at larger $t$.

Several studies have reported that the rate of decline in naïve T cell production is faster at young ages and slows as individuals get older (Steinmann 1986; Ritter and Palmer 1999; Mitchell et al. 2010), suggesting a power function (or hyperbolic function) of age. Tsukamoto et al. (2009) found that the increased lifespan of naïve T cells in old mice was associated with the proapoptotic molecule Bim, and that the function of these long-lived naïve T cells was defective. Den Braber et al. (2012) quantified the increased lifespan of naïve T cells in older mice and explained it by considering a density-dependent decay rate, since the number of naïve T cells decreases with age.

Thymic involution has traditionally been considered a deterioration of the immune system caused by aging and is often regarded as a typical example of immunosenescence. In the present paper, we proposed an alternative interpretation—namely, that it may represent an adaptive strategy of the host, balancing the need to generate acquired immunity against previously unencountered pathogens with the substantial cost of producing naïve T cells in the thymus. We developed an optimal control model to explore the outcomes expected if this interpretation is correct. We believe this hypothesis is worth investigating further, as it offers novel explanations and predictions regarding immune system dynamics. However, to test the accuracy of the model predictions presented in this paper, further empirical studies are needed, such as quantitative measurements of the number, diversity, production rate, and turnover time of naïve T cells in the periphery, potentially across different animal species. We hope this theoretical work will stimulate greater interest among immunologists.

## Authorship Contributions

YI: Conceptualization, Methodology, Formal analysis, Visualization, Writing – original draft. RH: Conceptualization, Numerical analysis, Visualization, Writing – original draft. AH: Conceptualization, Writing – original draft. KM: Conceptualization, Numerical analysis, Visualization, Writing – original draft.

## Figures and Tables

**Fig. 5:**
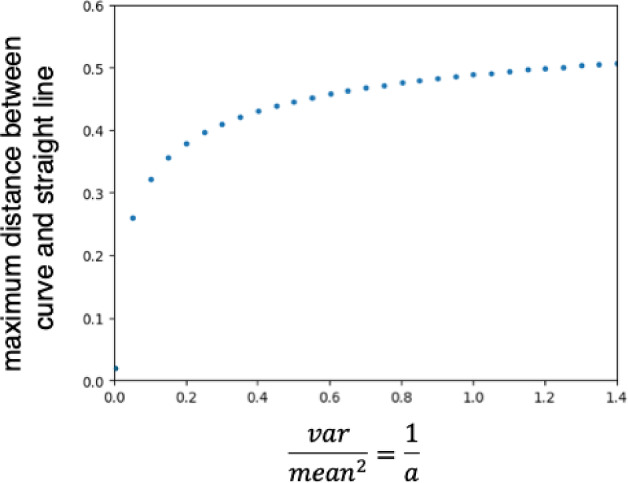
Relative magnitude the difference between the curve of $logh\left( t \right)$ and the straight line, corresponding to exponential decrease with age. The vertical axis indicates the relative difference, defined as Eq. (A.4b). The encounter rate $f$ follows a gamma distribution with the same mean $\overline{f} = a/b = 0.1$. The horizontal axis represents $variance/mean^{2} = 1/a$. The other parameter is: $Kun/m = 10^{4}$

**Fig. 6:**
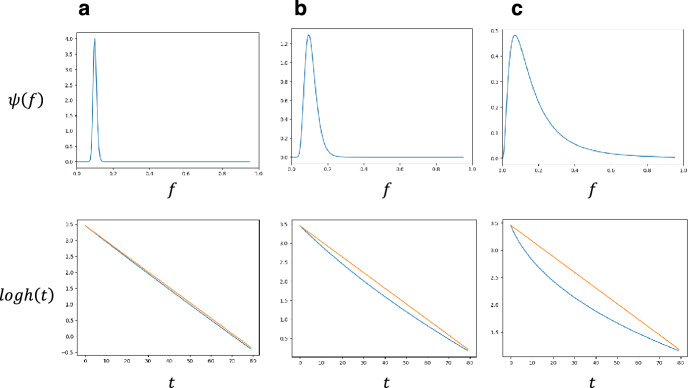
Optimal schedule of naïve T cell production $h\left( t \right)$ for different probability distribution of $f$. $\psi \left( f \right)$ follow log-normal distributions. Three cases, **a**, **b**, and **c**, have the same mean value $\overline{f} = e^{{\mu + \frac{1}{2}\sigma^{2} }} = 0.1$; but they differ in variance. Ratio, $variance/mean^{2} = e^{{\sigma^{2} }} - 1$, is as follows: **a** $0.01$; **b** $0.1$; **c** $1$. Three parts in each column correspond to those in Fig. 4 in the text. Parameters are: $Kn/m = 10^{4}$. **a** $\mu = - 2.30495, \sigma^{2} = 0.0099$; **b** $\mu = - 2.3475, \sigma^{2} = 0.0995$; and **c** $\mu = - 2.645, \sigma^{2} = 0.69$

**Fig. 7:**
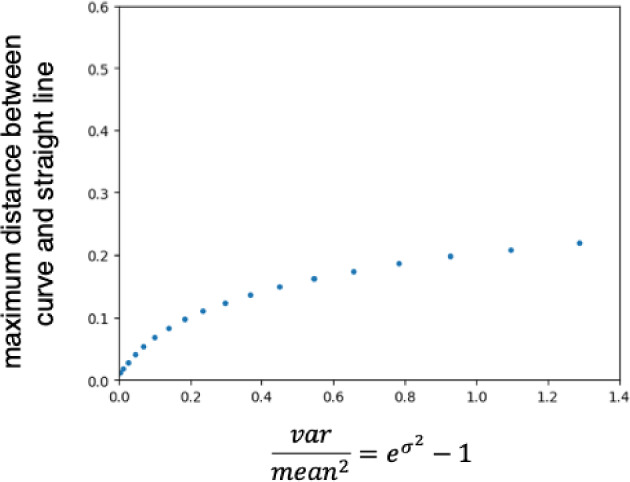
Relative magnitude of the difference between the curve of $logh\left( t \right)$ and the straight line. The value is defined by Eq. (A.4b). The horizontal axis represents $variance/mean^{2} = e^{{\sigma^{2} }} - 1$. The parameter $f$ among antigens is given by a log-normal distribution. Both $\mu$ and $\sigma^{2}$ changed with the mean fixed: $\overline{f} = e^{{\mu + \frac{1}{2}\sigma^{2} }} = 0.1$. $Kn/m = 10^{4}$

**Fig. 8:**
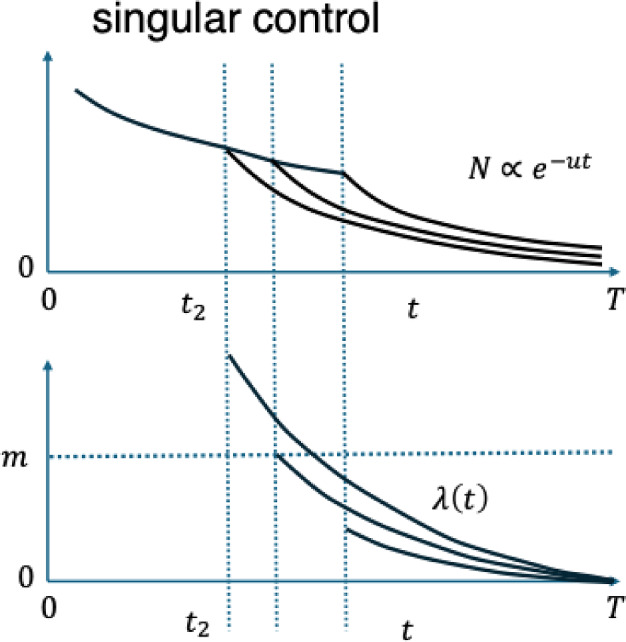
Iterative calculation to determine $t_{2}$. Refer to the text of Appendixes B and D for the explanation

**Fig. 9:**
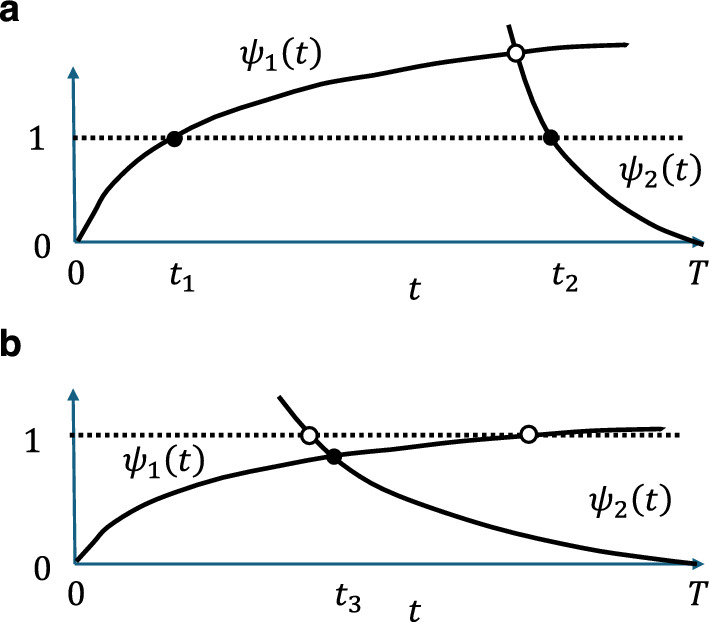
Illustration of the relationship between $t_{1}$, $t_{2}$ and $t_{3}$. Two curves represent $y = \psi_{1} \left( t \right)$ and $y = \psi_{2} \left( t \right)$. The horizontal line indicates $y =$ 1. **a** $t_{1} < t_{2}$ holds. The optimal schedule has the singular control between $t_{1}$ and $t_{2}$. **b** $t_{1} > t_{2}$ holds. The optimal schedule has no period of singular control. There is $t_{3}$, satisfuing $t_{1} > t_{3} > t_{2}$, and the switching between initial and final phases occurs at $t_{3}$

## Data Availability

Data sharing does not apply to this article, as no datasets were generated or analyzed during this study.

## Deviation from the Exponential Decrease in Naïve T Cell Production with Age

We here discuss the deviation from the exponential decline with age for observed curve of the production rate $h\left( t \right)$ versus age $t$. We plot the logarithm of the production rate $logh\left( t \right)$ versus age $t$. If $logh\left( t \right)$ shows a straight line with a negative slope, we conclude that it follows an exponential decrease: $h\left( t \right) \propto e^{ - at}$. We can measure the deviation from the exponential decrease by the deviation from the straight line.

From the general formula Eq. (4), we can derive $\frac{d}{dt}logh\left( t \right) < 0$ and $\frac{{d^{2} }}{{dt^{2} }}logh\left( t \right) > 0$ as follows:

From Eq. (4), we have

A.1 $$logh\left( t \right) = \frac{1}{2}log\frac{Ku}{m} + \frac{1}{2}log\mathop \sum \limits_{i = 1}^{n} f_{i} e^{{ - f_{i} t}}$$

The derivatives are:

A.2 $$\frac{d}{dt}logh\left( t \right) = \frac{1}{2}\frac{{\mathop \sum \nolimits_{i = 1}^{n} \left( { - 1} \right)f_{i}^{2} e^{{ - f_{i} t}} }}{{\mathop \sum \nolimits_{i = 1}^{n} f_{i} e^{{ - f_{i} t}} }} < 0$$

and

$$\frac{{d^{2} }}{{dt^{2} }}logh\left( t \right) = \frac{1}{2}\frac{{\mathop \sum \nolimits_{i = 1}^{n} f_{i} e^{{ - f_{i} t}} \mathop \sum \nolimits_{i = 1}^{n} f_{i}^{3} e^{{ - f_{i} t}} - \left( {\mathop \sum \nolimits_{i = 1}^{n} f_{i} e^{{ - f_{i} t}} } \right)^{2} }}{{\left( {\mathop \sum \nolimits_{i = 1}^{n} f_{i} e^{{ - f_{i} t}} } \right)^{2} }}$$

Now, since $f_{i}$ varies between strains, we have the following relation:

$$\mathop \sum \limits_{i = 1}^{n} \left( {f_{i} - \frac{{\mathop \sum \nolimits_{i = 1}^{n} f_{i}^{2} e^{{ - f_{i} t}} }}{{\mathop \sum \nolimits_{i = 1}^{n} f_{i} e^{{ - f_{i} t}} }}} \right)^{2} \frac{{f_{i} e^{{ - f_{i} t}} }}{{\mathop \sum \nolimits_{i = 1}^{n} f_{i} e^{{ - f_{i} t}} }} \ge 0$$

where the equality holds if and only when all $f_{i}$ are identical. This inequality is rewritten as follows

$$\mathop \sum \limits_{i = 1}^{n} f_{i} e^{{ - f_{i} t}} \mathop \sum \limits_{i = 1}^{n} f_{i}^{3} e^{{ - f_{i} t}} - \left( {\mathop \sum \limits_{i = 1}^{n} f_{i} e^{{ - f_{i} t}} } \right)^{2} \ge 0$$

Hence, we conclude

A.3 $$\frac{{d^{2} }}{{dt^{2} }}logh\left( t \right) \ge 0$$

The graph of $logh\left( t \right)$ versus $t$ should be a decreasing curve, with the steeper slope for smaller values of $t$. The deviation from the exponential decrease can be evaluated by the difference between the curve and a straight line.

## When ${\boldsymbol{f}}$ Follows a Gamma Distribution

The straight lines connect two points at age 0 and age 80 and are given as follows:

A.4a $$SL\left( t \right) = logh\left( 0 \right) - \frac{t}{80}\left( {logh\left( 0 \right) - logh\left( {80} \right)} \right)$$

We consider the maximum difference between the straight line and the curve as follows: $max_{0 < t < 80} \left[ {SL\left( t \right) - logh\left( t \right)} \right]$. We considered the relative magnitude of the maximum difference divided by $\left( {logh\left( 0 \right) - logh\left( {80} \right)} \right)$. It is defined as follows:

A.4b $$\left[ {\begin{array}{*{20}c} {\text{relative magnitude of deviation}} \\ {\text{from the straight line}} \\ \end{array} } \right] = \frac{{max_{0 < t < 80} \left[ {SL\left( t \right) - logh\left( t \right)} \right]}}{{logh\left( 0 \right) - logh\left( {80} \right)}}$$

In Figure 5. we plot the relative magnitude of deviation, given as Eq. (A.4b). The horizontal axis is $1/a$, ratio of variance to squared mean: $var/mean^{2}$. Figure 5 illustrates that as the variance increases, the curve of $logh\left( t \right)$ deviates more from the straight line, and the magnitude of difference increases with $1/a$.

## When ${\boldsymbol{f}}$ Follows Lognormal Distribution for Antigen-Abundance Relation

In ecology, a "community" refers to an assembly of different species that inhabit a specific area and coexist while interacting with one another. A commonly observed species-abundance relation in a well-studied community is a lognormal distribution (Preston 1948; Pielou 1966; Whittaker 1975). It states that the fraction of species with the logarithmic abundance $z = logf$ has a normal distribution with mean ${\upmu }$ and variance ${\upsigma }^{2}$. The probability distribution of $f = e^{z}$, instead of $logf = z$, is given as follows:

A.5 $$Pr\left[ {f \le \left[ {\text{Number of species}} \right] < f + df} \right] \approx \frac{1}{{\sqrt {2\pi \sigma^{2} } }}exp\left[ { - \frac{{\left( {logf - \mu } \right)^{2} }}{{2\sigma^{2} }}} \right]\frac{1}{f}df$$

This is $\psi \left( f \right)$, the probability distribution of $f$ between strains. The mean value of $f$ is ${\mathrm{e}}^{{{\upmu } + \frac{1}{2}{\upsigma }^{2} }}$ and the variance is ${\mathrm{e}}^{{2{\upmu } + {\upsigma }^{2} }} \left( {{\mathrm{e}}^{{{\upsigma }^{2} }} - 1} \right)$.

From Eq. (2), we have

$$h\left( t \right) = \sqrt {\frac{Kn}{{mu}} \cdot \frac{1}{n}\mathop \sum \limits_{i = 1}^{n} f_{i} e^{{ - f_{i} t}} } \approx \sqrt {\frac{Kn}{{mu}}} \cdot \sqrt {\mathop \smallint \limits_{0}^{\infty } fe^{ - ft} \frac{1}{{\sqrt {2\pi \sigma^{2} } }}exp\left[ { - \frac{{\left( {logf - \mu } \right)^{2} }}{{2\sigma^{2} }}} \right]\frac{1}{f}df}$$

The integral within the square root is rewritten as follows:

A.6 $$h\left( t \right) = \sqrt {\frac{Kn}{{mu}}} \cdot \sqrt {\mathop \smallint \limits_{ - \infty }^{\infty } e^{z} e^{{ - te^{z} }} \frac{1}{{\sqrt {2\pi \sigma^{2} } }}exp\left[ { - \frac{{\left( {z - \mu } \right)^{2} }}{{2\sigma^{2} }}} \right]dz}$$

where we set $z = logf.$

Figure 6 illustrates the optimal speed of naïve T cell supply from the thymus $h\left( t \right)$, shown in Eq. (A.6). Figure 6a, b, and c illustrate the probability distribution of $f$ (top parts) and the logarithmic value of $h\left( t \right)$, namely $logh\left( t \right)$ (bottom parts). They have the same mean values $\overline{f} = exp\left[ {\mu + \sigma^{2} /2} \right]$, but different variances. The optimal rate of naïve T cell supply $h\left( t \right)$ decreases with age $t$. When ${\upsigma }^{2}$ is small (Fig. 6a), the distribution of $f$ is sharpy centered around $\overline{f}$, and the optimal $h\left( t \right)$ is close to an exponential function, indicated by a straight line. As ${\upsigma }^{2}$ becomes larger (Fig. 6b, c), the distribution of $f$ has a larger variance, and $h\left( t \right)$ becomes more like a power function of age. The curve of $logh\left( t \right)$ becomes deviated from a straight line more strongly as variance ${\upsigma }^{2}$ increases.

Figure 7 indicates relative magnitude of the difference between the curve and the corresponding straight line, given by Eq. (A.4b). The line connects $\left( {0,logh\left( 0 \right)} \right)$ and $\left( {80,logh\left( {80} \right)} \right)$, and the maximum difference between $logh\left( t \right)$ and $SL\left( t \right)$ is divided by the difference, $logh\left( 0 \right) - logh\left( {80} \right)$. The relative magnitude of the difference increased with ratio $variance/mean^{2}$ of $f$.

## Appendix B

We consider a candidate solution to the control problem, as illustrated in Fig. 4a in the text. The top, middle, and bottom panels indicate the optimal schedule of naïve T cell production $h\left( t \right)$, the number of naïve T cell clones in the periphery $N\left( t \right)$, and the costate variable $\lambda \left( t \right)$, respectively. The horizontal axis is for age $t$ ($0 < t < T$). The solution consists of three qualitatively distinct phases. In contrast, Fig. 4b illustrates a case with two distinct phases. In this Appendix, we describe the procedure for deriving the optimal control.

## Three-Phase Solution: with Singular Control

We first focus the situation in which different strains have equal encounter rate: $f_{i} \approx \overline{f}$ ($i = 1,2,3,..,n$). Then $\sum\limits_{i = 1}^{n} {f_{i} e^{{ - f_{i} t}} \approx n\overline{f}e^{{ - \overline{f}t}} }$ holds. Later in Appendix C, we consider the case in which the strains vary with respect to $f_{i}$. As a candidate for the optimal control schedule, we consider the following three-phase solution:

Initial phase ($0 < t < t_{1}$): Naïve T cells are produced at the maximum speed.

Middle phase ($t_{1} < t < t_{2}$): Naïve T cells are produced at an intermediate spped. In this phase, singular control occurs.

Final phase ($t_{2} < t < T$): No naïve T cells is produced.

The solution of this type can be constructed first by noting the singular control in phase II ($t_{1} < t < t_{2}$). From Eq. (11) in the text, together with the singular control condition $\lambda \left( t \right) = m$, we have

B.1 $$0 = - \frac{K}{{N\left( t \right)^{2} }}\mathop \sum \limits_{i = 1}^{n} f_{i} e^{{ - f_{i} t}} + um$$

which is rewritten as

B.2 $$N\left( t \right) = \sqrt {\frac{K}{um}\mathop \sum \limits_{i = 1}^{n} f_{i} e^{{ - f_{i} t}} }$$

When all the strains have a common encounter rate $f_{i} \cong \overline{f}$, Eq. (B.2) becomes as follows:

B.3a $$N\left( t \right) = \sqrt {\frac{{Kn\overline{f}}}{um}} e^{{ - \frac{1}{2}\overline{f}t}}$$

From Eqs. (2) and (B.3a), we have the rate of production of naïve T cells from the thymus as follows:

$$h\left( t \right) = \frac{dN}{{dt}} + uN = \left( {u - \frac{{\overline{f}}}{2}} \right)\sqrt {\frac{{Kn\overline{f}}}{um}} e^{{ - \frac{{\overline{f}}}{2}t}}$$

Which are the equations governing the singular control.

Next, to determine the end time for the middle phase, we analyze the solution during the final phase. In the final phase $\left( {t_{2} < t < T} \right)$, the following dynamics hold for state and costate variables:

B.4a $$\frac{dN}{{dt}} = - uN\left( t \right)$$

B.4b $$\frac{d\lambda }{{dt}} = - \frac{{Kn\overline{f}}}{{N\left( t \right)^{2} }}e^{{ - \overline{f}t}} + u\lambda \left( t \right)$$

respectively. Hence, we have $N\left( t \right) = N\left( {t_{2} } \right)e^{{ - u\left( {t - t_{2} } \right)}}$ for $t_{2} < t < T$. By integrating Eq. (B.4b) with terminal condition $\lambda \left( T \right) = 0$, the solution for the costate variable is given as follows:

B.5 $$\lambda \left( t \right) = \mathop \smallint \limits_{t}^{T} \frac{{Kn\overline{f}}}{{N\left( {t^{\prime } } \right)^{2} }}e^{{ - \overline{f}t^{\prime } }} e^{{ - u\left( {t^{\prime } - t} \right)}} dt^{\prime } \;for\;t_{2} < t < T$$

At the boundary between middle and final phases, $\lambda \left( {t_{2} } \right) = m$ holds, because $\lambda \left( t \right)$ is continuous and $\lambda \left( t \right) = m$ holds in the middle phase. Hence, we have

B.6 $$\begin{aligned} m = & \lambda \left( {t_{2} } \right) = \mathop \smallint \limits_{{t_{2} }}^{T} \frac{{Kn\overline{f}}}{{\left[ {N\left( {t_{2} } \right)e^{{ - u\left( {t^{\prime } - t_{2} } \right)}} } \right]^{2} }}e^{{ - \overline{f}t^{\prime } }} e^{{ - u\left( {t^{\prime } - t_{2} } \right)}} dt^{\prime } \\ & \quad = \mathop \smallint \limits_{{t_{s} }}^{T} \frac{{Kn\overline{f}}}{{\frac{{Kn\overline{f}}}{um}e^{{ - \overline{f}t_{2} }} e^{{ - 2u\left( {t^{\prime } - t_{2} } \right)}} }}e^{{ - \overline{f}t^{\prime } }} e^{{ - u\left( {t^{\prime } - t_{2} } \right)}} dt^{\prime } \\ \end{aligned}$$

where we used Eq. (B.3a). Eq. (B.6) is rewritten as follows:

B.7 $$1 = u\mathop \smallint \limits_{{t_{2} }}^{T} \frac{{e^{{ - \overline{f}\left( {t^{\prime} - t_{2} } \right)}} }}{{e^{{ - u\left( {t^{\prime} - t_{2} } \right)}} }}dt^{\prime} = \frac{{u\left( {e^{{\left( {u - \overline{f}} \right)\left( {T - t_{2} } \right)}} - 1} \right)}}{{u - \overline{f}}}$$

which is rewritten as

B.8 $$T - t_{2} = \frac{1}{{u - \overline{f}}}log\left( {2 - \frac{{\overline{f}}}{u}} \right)$$

which is the same as Eq. (15) in the main text. Note that $0 < t_{2} < T$ holds.

Eq. (B.8) indicates that, as $u$ increases, $T - t_{2}$ decreases, and final phase becomes shorter.

Finally, we consider the initial phase (phase I), in which naïve T cells are produced at the fastest rate: $h\left( t \right) = h_{max}$. From differential equation Eq. (2) and the initial condition $N\left( 0 \right) = 0$, we h.

ave the following solution:

B.9 $$N\left( t \right) = \frac{{h_{max} }}{u}\left( {1 - e^{{ - ut_{1} }} } \right)\;for\; 0 < t < t_{1}$$

At the end of phase I, the number of naïve T cell clones in the periphery reaches the value specified by the singular control given by Eq. (B.3a). From these, we have

B.10 $$\frac{{h_{max} }}{u}\left( {1 - e^{{ - ut_{1} }} } \right) = \sqrt {\frac{{Kn\overline{f}}}{um}} e^{{ - \frac{{\overline{f}}}{2}t_{1} }}$$

Eq. (B.10) is the equation to determine $t_{1}$. Note that $t_{1} > 0$ holds.

The value of $t_{1}$ is calculated from Eq. (B.10) and that of $t_{2}$ is from Eq. (B.8). If they satisfy $t_{1} < t_{2}$, we can construct the three-phase solution, as illustrated in Fig. 4a of the text. For $t$ between $t_{1}$ and $t_{2}$, the optimal control is singular.

Here, we note that the calculation of the optimal control schedule often requires iterative calculations, as illustrated by Fig. 8. For example, the procedure to obtain the switching date $t_{2}$ through iterative calculation is as follows: [1] First we choose a trial value of $t_{2}$. [2] Using $t_{2}$, we calculate $N\left( {t_{2} } \right) = \sqrt {\frac{{Kn\overline{f}}}{um}} e^{{ - \frac{1}{2}\overline{f}t_{2} }}$ from the value along the singular control in the middle phase. [3] Then, we obtain the values of $N\left( t \right)$ in the final phase: $N\left( t \right) = \sqrt {\frac{{Kn\overline{f}}}{um}} e^{{ - \frac{1}{2}\overline{f}t_{2} }} e^{{ - u\left( {t - t_{2} } \right)}}$ (for $t_{2} < t < T$). [4] Next, we integrate the differential equation of $\lambda \left( t \right)$, given by (B.4b), backward from $T$, and it is given by Eq. (B.5). [5] We then calculate $\lambda \left( {t_{2} } \right)$. If this is close to $m$ (i.e., $\lambda \left( {t_{2} } \right) \approx m$), the value of $t_{2}$ can be regarded a the optimal one. [6] If $\lambda \left( {t_{2} } \right) > m$, we increase $t_{2}$; and if $\lambda \left( {t_{2} } \right) < m$, we decrease $t_{2}$. Then repeat this procedure [1]-[6] again.

Here, we note that $\lambda \left( {t_{2} } \right)$ decreases with $t_{2}$ by the following argument (Refer to Fig. 8). Because the rate of decrease in the middle phase is smaller than that in the final phase $\left( {\frac{1}{2}\overline{f} < u} \right)$, a larger $t_{2}$ makes $N\left( t \right)$ larger in the final phase. Since $N\left( t \right)$ exists in the denominator of the integrant of Eq. (B.5), a larger $N\left( t \right)$ makes $\lambda \left( {t^{\prime}} \right)$ smaller where $t^{\prime} < t$. In addition, a larger $t_{2}$ makes the interval between $t_{2}$ and $T$ narrower. Hence, a larger choice of $t_{2}$ makes the value of $\lambda \left( {t_{2} } \right)$ smaller, as illustrated in Fig. 8).

From this argument, if $\lambda \left( {t_{1} } \right) > m$, there exists a single $t_{2} > 0$ that satisfies $\lambda \left( {t_{1} } \right) = m$ (Fig. ^9^a). Instead, if $\lambda \left( {t_{1} } \right) < m$ (Fig. 9b). Then, we do not have the optimal control schedule with a singular-control phase, which will be discussed in the next subsection.

For the current problem, this iterative calculation is unnecessary, and we can obtain Eq. (B.8), which is the same as Eq. (15) in the text, from which we can determine $t_{2}$. However, this holds only when $\sum\limits_{i = 1}^{n} {f_{i} e^{{ - f_{i} t}} }$ is approximated by a single exponential function. In general cases, we must adopt the iterative calculation to determine the switching date $t_{2}$.

## Two-Phase Solution Without Singular Control

If $t_{1}$ and $t_{2}$ obtained from Eqs. (B.10) and (B.8) satisfy $t_{1} \ge t_{2}$, we do not have the optimal schedule including singular control. Then we have the two-phase solution given as follows:

Initial phase ($0 < t < t_{3}$) Production rate of naïve T cells is the highest:

B.11a $$h\left( t \right) = h_{max}$$

In this phase, $N\left( t \right) = \frac{{h_{max} }}{u}\left( {1 - e^{ - ut} } \right)$ holds.

Final phase $\left( {t_{3} < t < T} \right)$ No naïve T cell is produced.

B.11b $$h\left( t \right) = 0$$

In this phase, $N\left( t \right) = \frac{{h_{max} }}{u}\left( {1 - e^{{ - ut_{3} }} } \right)e^{{ - u\left( {t - t_{3} } \right)}}$ holds.

Let $t_{3}$ be the date for switching between phases I and III. From Eq. (12) in the text, the optimal choice of switching date $t_{3}$ can be determined by the condition for costate variable,

B.12a $$\lambda \left( t \right) > m,\;for\;0 < t < t_{3}$$

B.12b $$\lambda \left( {t_{3} } \right) = m,\;at\;t = t_{3}$$

B.12c $$\lambda \left( t \right) < m,\;for\;t_{3} < t < T$$

Please note that $\lambda \left( t \right) = m$ holds only at a single time point $t_{3}$: there is no period of singular control. The costate variable at $t_{3}$ is obtained in the same manner as the procedure for calculating $t_{2}$, given by Eq. (B.6).

B.13 $$\begin{aligned} m = & \lambda \left( {t_{3} } \right) = \mathop \smallint \limits_{{t_{3} }}^{T} \frac{{Kn\overline{f}}}{{\left[ {N\left( {t_{3} } \right)e^{{ - u\left( {t^{\prime } - t_{3} } \right)}} } \right]^{2} }}e^{{ - \overline{f}t^{\prime}}} e^{{ - u\left( {t^{\prime } - t_{3} } \right)}} dt^{\prime } \\ & \quad = \mathop \smallint \limits_{{t_{3} }}^{T} \frac{{Kn\overline{f}}}{{\left[ {\frac{{h_{max} }}{u}\left( {1 - e^{{ - ut_{3} }} } \right)} \right]^{2} e^{{ - 2u\left( {t^{\prime } - t_{3} } \right)}} }}e^{{ - \overline{f}t^{\prime } }} e^{{ - u\left( {t^{\prime } - t_{3} } \right)}} dt^{\prime } \\ & \quad = \frac{{Kn\overline{f}e^{{ - \overline{f}t_{3} }} }}{{\left[ {\frac{{h_{max} }}{u}\left( {1 - e^{{ - ut_{3} }} } \right)} \right]^{2} }}\mathop \smallint \limits_{{t_{3} }}^{T} \frac{{e^{{ - \overline{f}\left( {t^{\prime } - t_{3} } \right)}} }}{{e^{{ - u\left( {t^{\prime } - t_{3} } \right)}} }}dt^{\prime } \\ \end{aligned}$$

which is rewritten as follows:

B.14 $$\left[ {\frac{{h_{max} }}{u}\left( {1 - e^{{ - ut_{3} }} } \right)} \right]^{2} = \frac{{Kn\overline{f}e^{{ - \overline{f}t_{3} }} }}{m} \cdot \frac{{e^{{\left( {u - \overline{f}} \right)\left( {T - t_{3} } \right)}} - 1}}{{u - \overline{f}}}$$

The solution $t_{3}$ of Eq. (B.14) provides the time for the end of phase I with the fastest naïve T cell production and the start of phase III.

## The Condition for a Singular Solution to Be a Part of the Optimal Schedule

Now we consider the relationship of switching date $t_{3}$ for the two-phase solution and two switching dates $t_{1}$ and $t_{2}$ for the three-phase solution. Eqs. (B.10) and (B.7) provide the conditions for $t_{1}$ and $t_{2}$, respectively. We define $\psi_{1} \left( t \right)$ as a monotonically increasing function of $t$:

B.15a $$\psi_{1} \left( t \right) = \frac{{h_{max} }}{u}\left( {1 - e^{{ - ut_{1} }} } \right)\sqrt {\frac{um}{{Kn\overline{f}}}} e^{{\frac{{\overline{f}}}{2}t_{1} }}$$

and $\psi_{2} \left( t \right)$ as a monotonically decreasing function of $t$:

B.15b $$\psi_{2} \left( t \right) = \sqrt {u\frac{{e^{{\left( {u - \overline{f}} \right)\left( {T - t_{2} } \right)}} - 1}}{{u - \overline{f}}}}$$

Eqs. (B.10) indicates that $t_{1}$ is a solution of $\psi_{1} \left( {t_{1} } \right) = 1$, and Eq. (B.7) indicates that $t_{2}$ is a solution of $\psi_{2} \left( {t_{2} } \right) = 1$. They satisfy $t_{1} > 0$ and $t_{2} < T$. Additionally, if they satisfy $t_{1} < t_{2}$, there exists the three-phase solution, which includes the initial phase (from 0 to $t_{1}$), the middle phase (from $t_{1}$ to $t_{2}$), and the final phase (from $t_{2}$ to $T$).

On the other hand, Eq. (B.14) that determine $t_{3}$ is rewritten as follows:

B.15c $$\frac{{h_{max} }}{u}\left( {1 - e^{{ - ut_{3} }} } \right)\sqrt {\frac{um}{{Kn\overline{f}}}} e^{{\frac{{\overline{f}}}{2}t_{3} }} = \sqrt {u \cdot \frac{{e^{{\left( {u - \overline{f}} \right)\left( {T - t_{3} } \right)}} - 1}}{{u - \overline{f}}}}$$

which implies that $t_{3}$ is the solution of the following equality: $\psi_{1} \left( {t_{3} } \right) = \psi_{2} \left( {t_{3} } \right)$.

Figure 9a and b illustrate two curves for the two functions ($$\psi_{1} \left( t \right)$$ and $$\psi_{2} \left( t \right)$$) and a horizontal line of height 1. If $$\psi_{1} \left( {t_{3} } \right) = \psi_{2} \left( {t_{3} } \right) > 1$$, the three values satisfy $$t_{1} < t_{3} < t_{2}$$ (Fig. 9a), in which there is an interval for singular control (middle phase; for $$t_{1} < t < t_{2}$$). In contrast, if $$\psi_{1} \left( {t_{3} } \right) = \psi_{2} \left( {t_{3} } \right) < 1$$, three values satisfy $$t_{2} < t_{3} < t_{1}$$ (Fig. 9b), and there is no interval for singular control (no middle phase): the optimal schedule consists initial phase ($$0 < t < t_{3}$$) and final phase ($$t_{3} < t < T)$$.

## The Second Order Condition for Optimality of a Singular Control

This condition is called the generalized Legendre-Clebsch condition (Kelley et al. 1967; Robbins 1967; Iwasa and Roughgarden 1984; Iwasa et al. 2025). We confirm the following inequality along the singular control:

C.1 $$\frac{\partial }{\partial h}\frac{{d^{2} }}{{dt^{2} }}\left( {\frac{\partial H}{{\partial h}}} \right) > 0.$$

This can be calculated as follows:

C.2 $$\begin{gathered} \frac{\partial H}{{\partial h}} = - m + \lambda \left( t \right) \hfill \\ \frac{d}{dt}\frac{\partial H}{{\partial h}} = \frac{d\lambda }{{dt}} = - \frac{\partial H}{{\partial N}} = - \frac{K}{{N^{2} }}\mathop \sum \limits_{i = 1}^{n} f_{i} e^{{ - f_{i} t}} + \lambda u \hfill \\ \frac{{d^{2} }}{{dt^{2} }}\frac{\partial H}{{\partial h}} = \frac{d}{dt}\left( { - \frac{K}{{N^{2} }}\mathop \sum \limits_{i = 1}^{n} f_{i} e^{{ - f_{i} t}} + \lambda u} \right) \hfill \\ = \frac{2K}{{N^{3} }}\left( {h - uN} \right)\mathop \sum \limits_{i = 1}^{n} f_{i} e^{{ - f_{i} t}} + \frac{K}{{N^{2} }}\mathop \sum \limits_{i = 1}^{n} f_{i}^{2} e^{{ - f_{i} t}} + \left( { - \frac{K}{{N^{2} }}\mathop \sum \limits_{i = 1}^{n} f_{i} e^{{ - f_{i} t}} + \lambda u} \right)u \hfill \\ \end{gathered}$$

The first term in the right-hand side of Eq. (C.2) includes $h$, but none of the others includes $h$. Hence, we have

C.3 $$\frac{\partial }{\partial h}\frac{{d^{2} }}{{dt^{2} }}\left( {\frac{\partial H}{{\partial h}}} \right) = \frac{2K}{{N^{3} }}\mathop \sum \limits_{i = 1}^{n} f_{i} e^{{ - f_{i} t}} > 0$$

Hence, we can conclude that the singular control satisfies the second-order condition for the optimality.

## Appendix D

Here, we consider the case when the pathogen strains differ in the frequency of encounter with the host. When the distribution of $f_{i}$ ($i = 1,2,...,n$) is approximated by a probability distribution of $f$ with a continuous probability density, $\psi \left( f \right)$, we have $\frac{1}{n}\mathop \sum \limits_{i = 1}^{n} f_{i} e^{{ - f_{i} t}} \approx \mathop \smallint \limits_{0}^{\infty } fe^{ - ft} \psi \left( f \right)df$. We here consider the case with a Gamma distribution: $\psi \left( f \right) = \frac{{f^{a - 1} e^{ - bf} }}{{{\Gamma }\left( a \right)\frac{1}{{b^{a} }}}}$, which indicates a probability density either with a single peak or a monotonically decreasing density. It has mean $\overline{f} = \frac{a}{b}$ and variance $\frac{a}{{b^{2} }}$.

D.1 $$\frac{1}{n}\mathop \sum \limits_{i = 1}^{n} f_{i} e^{{ - f_{i} t}} \approx \mathop \smallint \limits_{0}^{\infty } fe^{ - ft} \frac{{f^{a - 1} e^{ - bf} }}{{{\Gamma }\left( a \right)\frac{1}{{b^{a} }}}}df = \overline{f}\frac{1}{{\left( {1 + \overline{f}t\frac{1}{a}} \right)^{a + 1} }}$$

where we replaced $b$ by $a/\overline{f}$. The inverse of parameter $a$ indicates the ratio of variance to squared mean: $\frac{1}{a} = \frac{{{\mathrm{variance}}}}{{{\mathrm{mean}}^{2} }}$. If $a$ is infinitely large, $\psi \left( f \right)$ becomes sharply concentrated at the mean $\overline{f}$, and Eq. (D.1) becomes $\overline{f}e^{{ - \overline{f}t}}$, which is the case examined in Appendix B. In the following we examine the effect of variance in $f$ to the optimal schedule of naïve T cell production.

## Optimal Schedule with Singular Control

We consider a three-phase solution that begins with the initial phase ($0 < t < t_{1}$), corresponding to the rapid production of naïve T cells immediately after birth, followed by the middle phase ($t_{1} < t < t_{2}$), corresponding a singular control with both $N\left( t \right)$ and $h\left( t \right)$ positive and decrease with age. The final phase ($t_{2} < t < T$) indicates a period of no further production of naïve T cells.

We first calculate the singular control in the middle phase ($t_{1} < t < t_{2}$). Equation $\lambda \left( t \right) = m$ holds, as it makes the coefficient of control variable $h\left( t \right)$ to zero. From the differential equation of $\lambda \left( t \right)$, given by Eq. (11) in the text, we have

D.2 $$0 = - \frac{{Kn\overline{f}}}{{N\left( t \right)^{2} }} \cdot \frac{1}{{\left( {1 + \overline{f}t\frac{1}{a}} \right)^{a + 1} }} + mu$$

which becomes

D.3 $$N\left( t \right) = \sqrt {\frac{{Kn\overline{f}}}{mu}} \frac{1}{{\left( {1 + \overline{f}t\frac{1}{a}} \right)^{{\frac{a + 1}{2}}} }}\;{\mathrm{for}}\;t_{1} < t < t_{2}$$

From the Eq. (2) in the text, we have $h\left( t \right) = \frac{dN}{{dt}} + uN$ which leads to

D.4 $$h\left( t \right) = \sqrt {\frac{{Kn\overline{f}}}{mu}} \left( {u - \frac{{\frac{a + 1}{2}}}{{1 + \overline{f}t\frac{1}{a}}}} \right)\frac{1}{{\left( {1 + \overline{f}t\frac{1}{a}} \right)^{{\frac{a + 1}{2}}} }}\;{\mathrm{for}}\;t_{1} < t < t_{2}$$

For simplicity, we here consider the case $u > \frac{a + 1}{2}$ holds. Then, the second factor of Eq. (D.4) is positive for all $t$, and we can construct three-phase solution: At birth (initial phase), a very high production of naïve T cells should be done to make the starting value of $N\left( t \right)$ in the beginning of singular control. For the final phase ($t_{2} < t < T$), we have $h\left( t \right) = 0$. The number of naïve T cell clones in the periphery decreases exponentially with age $t$.

D.5 $$N\left( t \right) = \sqrt {\frac{{Kn\overline{f}}}{mu}} \frac{1}{{\left( {1 + \overline{f}t_{2} \frac{1}{a}} \right)^{{\frac{a + 1}{2}}} }}e^{{ - u\left( {t - t_{2} } \right)}}$$

Hence, if we know the optimal value for switching date $t_{2}$, we can determine $N\left( t \right)$ and $h\left( t \right)$ from Eqs. (D.3), (D.4), and (D.5). However, the procedure of obtaining the switching date $t_{2}$ is much more complex than when all that $f_{i}$ are the same, explained in Appendix B. We explain this in the following subsection.

## Iterative Calculation for Determining the Optimal Switching Date: ${\boldsymbol{t}}_{2}$

Here we would like to explain the procedure of iterative calculation as follows:

[1] First we choose a trial value of $t_{2}$.

[2] Using $t_{2}$, we calculate $N\left( {t_{2} } \right) = \sqrt {\frac{{Kn\overline{f}}}{mu}} \frac{1}{{\left( {1 + \frac{{\overline{f}}}{a}t_{2} } \right)^{{\frac{a + 1}{2}}} }}$ from the value along the singular control in phase II.

[3] Then, we obtain the values of $N\left( t \right)$ in the phase III as $N\left( t \right) = N\left( {t_{2} } \right)e^{{ - u\left( {t - t_{2} } \right)}}$, for $t_{2} < t < T$, this is the same as Eq. (D.5).

[4] Finally, we integrate the differential equation of $\lambda \left( t \right)$ given by the following:

D.6 $$\frac{d\lambda }{{dt}} = - \frac{{Kn\overline{f}}}{{N\left( t \right)^{2} }}\left( t \right) + u\lambda \left( t \right)$$

backward from the final time $T$.

[5] We then calculate $\lambda \left( {t_{2} } \right)$. If this is close to $m$ (i.e., $\lambda \left( {t_{2} } \right) \approx m$), the chosen value of $t_{2}$ can be regarded the optimal value.

[6] If $\lambda \left( {t_{2} } \right) > m$, we increase $t_{2}$; and if $\lambda \left( {t_{2} } \right) < m$, we decrease $t_{2}$. Then repeat this procedure [1]-[6] again.

Figure 8 illustrate the process [6] above. Here, we note that $\lambda \left( {t_{2} } \right)$ decreases with $t_{2}$ by the following argument: Because the rate of decrease in phase II is smaller than that in phase III, a larger $t_{2}$ makes $N\left( t \right)$ larger in phase III. Since $N\left( t \right)$ exists in the denominator of the integrant of Eq. (A.4), a larger $N\left( t \right)$ makes $\lambda \left( {t^{\prime}} \right)$ smaller where $t^{\prime} < t$. In addition, a smaller $t_{2}$ makes the interval $t_{2}$ and $T$ wider. Hence, the value of $\lambda \left( {t_{2} } \right)$ becomes smaller for a larger choice of $t_{2}$. lf the costate variable at time $t_{2}$ is close to $m$ (i.e., $\lambda \left( {t_{2} } \right) \approx m$), we can regard $t_{2}$ as the optimal date of switching between two phases (Refer to Fig. 5). If not, we adjust $t_{2}$ and repeat this procedure. Such an iterative procedure is common in dynamic optimization using the Pontryagin’s maximum principle (i.e., Iwasa et al. 2025).

This iterative calculation was unnecessary when the time dependence of the Hamiltonian includes an exponential function of $t$, which was a fortunate exception. However, when the Hamiltonian includes a power function of $t$, we need to adopt the iterative procedure to search for the optimal switching date.

## When the Turnover of Peripheral Naïve T Cells is Slow

If the turnover of peripheral population is very slow ($u$ is very small), the value of $t_{2}$ calculated in the manner explained above turned out to be $t_{2} \le t_{1}$, and the candidate solution for the control problem has no phase of singular control. The optimal solution is the fastest production of naïve T cells in the initial phase and then switched to the final phase of no production.

If the decay rate is slow and of a similar magnitude to the encounter rates of the pathogen strains, there exists a singular solution. We may consider the candidate for the singular solution as $N\left( t \right)$, given by Eq. (D.3). The corresponding rate of naïve T cell production rate $h\left( t \right)$, given by Eq. (D.4), may not be positive. In this section, $N\left( t \right)$ is a power function whose rate of exponential rate of decrease is rapid for smaller $t$ and slower for larger $t$. Consequently, the function $h\left( t \right) = \frac{dN}{{dt}} + uN$ may be negative for small $t$ and positive for large $t$, which occurs when $u < \frac{a + 1}{2}$. This suggests that the singular control phase begins at an intermediate age and ends at the onset of the final phase. The construction of the optimal solution is therefore more complex.

Note that this complication does not arise if all pathogen strains have the rate of encounter, as in the Appendix B, $N\left( t \right)$ is an exponential function, and the corresponding formula for $h\left( t \right)$ is also an exponential function, multiplied by a factor $u - \frac{1}{2}\overline{f}$. Depending on whether this factor is positive or negative, the singular solution can or cannot be constructed.
